# Supplementary material for: Establishing microbial composition measurement standards with reference frames
Source: Nat Commun. 2019 Jun 20;10:2719. doi: 10.1038/s41467-019-10656-5 (PMC6586903; doi:10.1038/s41467-019-10656-5)
Supplement: Supplementary file 1 — Supplementary Information [file 41467_2019_10656_MOESM1_ESM.pdf]

Supplementary Information

Establishing microbial composition measurement standards with reference frames

Morton and Marotz et al.

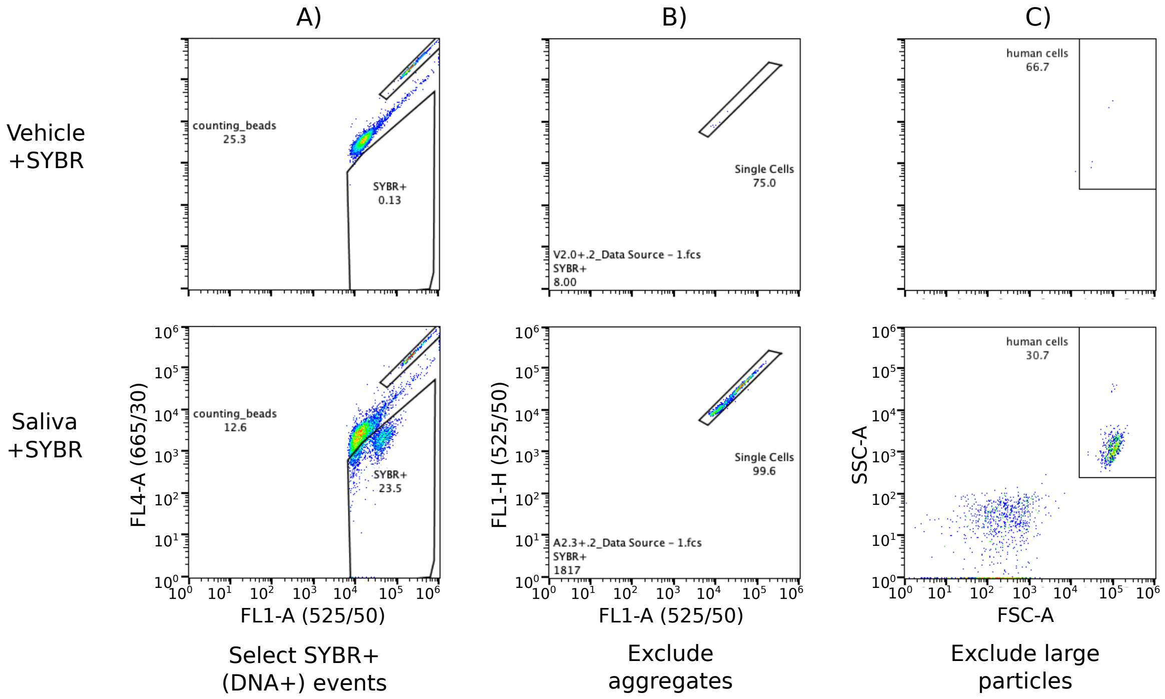

Supplementary Figure 1. Flow cytometry gating strategy. Microbial load was quantified using a protocol adapted from previously published work (as described in Methods). Vehicle controls (1x PBS) and filtered, diluted saliva samples were stained with 0.1x SYBR Green. A set volume of AccuCount Fluorescent Particles was added to each sample immediately prior to running). An initial gate was drawn to select SYBR-DNA+ events on a plot of FL1 (525 nm) vs FL4 (665 nm), since SYBR Green I increases fluorescence specifically in the FL1 channel upon intercalation in DNA. AccuCount bead particles, which are highly fluorescent across both channels, were also gated on this plot. B) Aggregates were excluded from the SYBR-DNA+ events by excluding events not along a linear axis on a plot of FL1-height versus FL1-area. C) Lastly, large particles were excluded by removing outliers on a plot of side scatter (SSC) versus forward scatter (FSC). The remaining events were used to estimate microbial concentration by comparing to the number of AccuCount Fluorescent Particles detected in the same amount of time, according to the manufacturer's instructions.

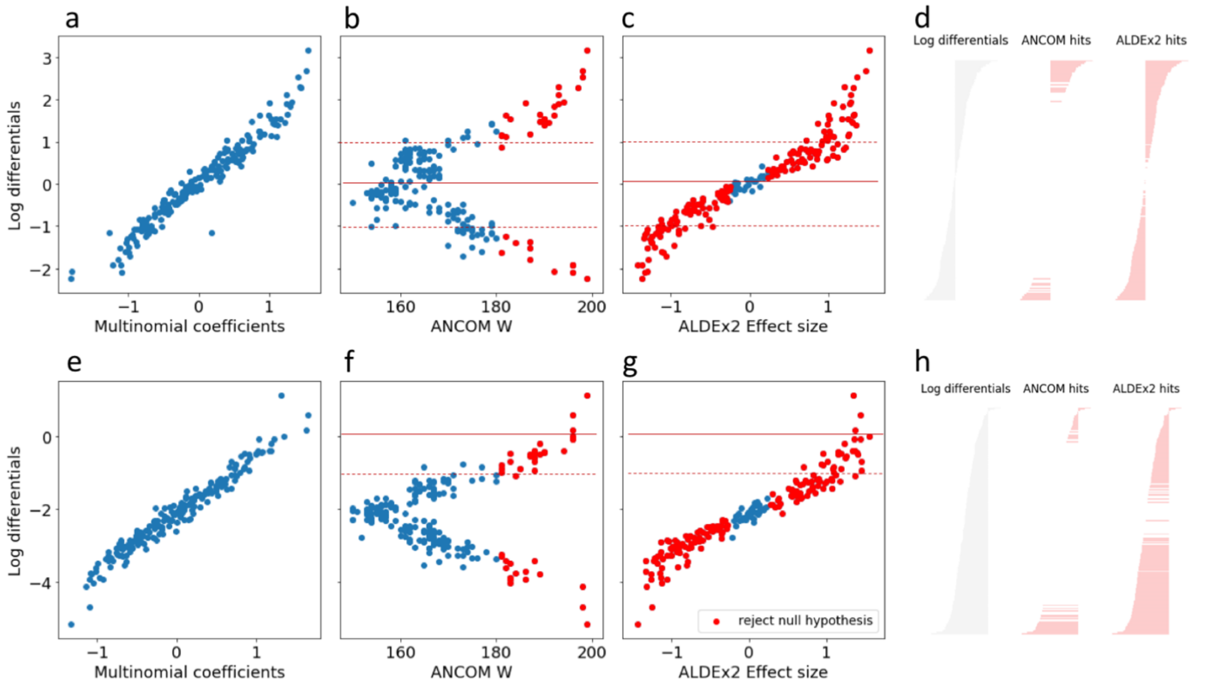

Supplementary Figure 2. Simulation benchmarks of multinomial regression, ANCOM and ALDEx2. (a-d) Comparison of the three tools when the microbial differences are centered around zero, with microbes that rejected the respective null hypothesis highlighted in red. (e-h) Comparison of the three tools when the microbial differences are centered around -2, with microbes that rejected the respective null hypothesis highlighted in red.

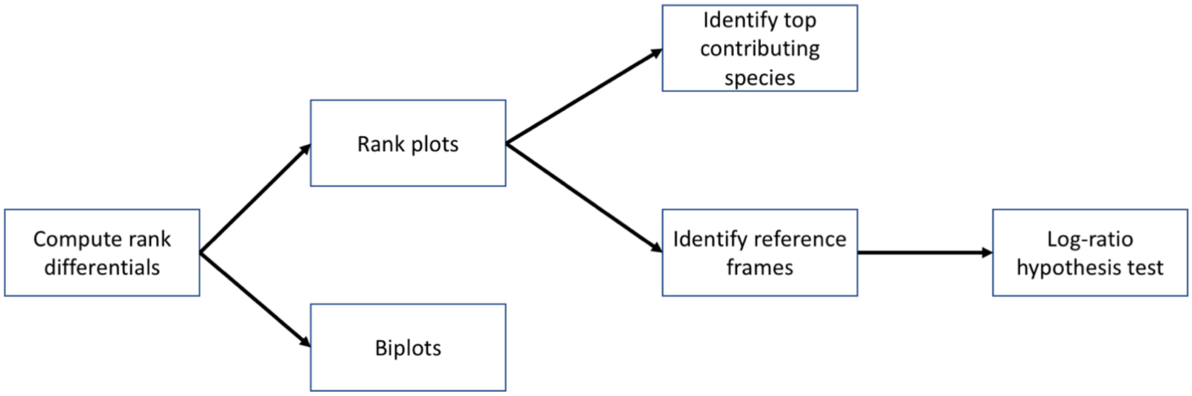

Supplementary Figure 3. Visualization of the recommended pipeline procedure for identifying and testing for differentially abundant microbes in the absence of absolute abundance data.
